# A Dose-Image Optimization Trial for Voluven®-Assisted Indocyanine Green Fluorescence-Guided Breast Cancer Sentinel Lymph Node Surgery

**DOI:** 10.1245/s10434-025-17696-w

**Published:** 2025-06-23

**Authors:** Yung-Chun Hsieh, Chiun-Sheng Huang, Yang-Hsiang Chan

**Affiliations:** 1 Department of Surgery, National Taiwan University Hospital Hsinchu Branch, Hsinchu, Taiwan R.O.C.; 2 Department of Surgery, National Taiwan University Hospital, Taipei, Taiwan R.O.C.; 3 National Taiwan University College of Medicine, Taipei, Taiwan R.O.C.; 4 Department of Applied Chemistry, National Yang Ming Chiao Tung University, Hsinchu, Taiwan R.O.C.; 5 Center for Emergent Functional Matter Science, National Yang Ming Chiao Tung University, Hsinchu, Taiwan R.O.C.; 6 Department of Medicinal and Applied Chemistry, Kaohsiung Medical University, Kaohsiung, Taiwan R.O.C.

**Keywords:** Indocyanine green, Sentinel lymph node biopsy, Breast cancer, Fluorescence-guided surgery, 6% hydroxyethyl starch, Dose optimization

## Abstract

**Background:**

Sentinel lymph node biopsy (SLNB) is critical in breast cancer staging, and indocyanine green (ICG) has emerged as a promising fluorescent tracer. Optimizing ICG concentration with an appropriate solvent such as Voluven® could improve imaging quality and SLN detection, yet the ideal protocol remains undefined. This study investigates the optimal ICG:Voluven concentration for SLNB in breast cancer surgery.

**Patients and Methods:**

In a prospective trial (April 2022–June 2023), 12 women with early breast cancer underwent SLNB with ICG:Voluven at 0.5 mg/mL (5×, *n* = 3), 0.25 mg/mL (10×, *n* = 6), or 0.125 mg/mL (20×, *n* = 3). Outcomes included SLN retrieval, signal-to-background ratio (SBR), areola-to-axilla traveling time (AAT), safety, and cost, assessed via Stryker SPY Portable Handheld Imaging System.

**Results:**

The 10× group (0.25 mg/mL) showed the highest median SBR (127.4, range 90.9–256.0) versus 5× (26.3, 2.7–133.2) and 20× (39.1, 5.3–98.4), retrieving three SLNs per patient consistently, unlike fewer in other groups. The 20× group had the shortest AAT (44.3 s) but lower SBR and procedural issues (e.g., subcutaneous dissection). The 5× group had the longest AAT (144.3 s) and reduced SLN detection. No adverse events occurred. The equivalent drug cost was around 1.5 US dollars per patient.

**Conclusions:**

The 0.25 mg/mL ICG:Voluven concentration offers an optimal balance of fluorescence imaging quality, SLN detection, and procedural efficiency for SLNB in breast cancer surgery. Its safety, effectiveness, and low cost make it a practical choice, especially in resource-limited settings. Larger studies are needed to validate these results and refine the protocol further.

Intraoperative fluorescence imaging has become an increasingly popular technique in precision surgery due to its superior resolution, signal-to-background ratio (SBR), safety, and ability for real-time intraoperative use. Indocyanine green (ICG), a commonly used infrared contrast agent since the introduction of silicon-based charge-coupled device (CCD) sensors for medical imaging, has been widely adopted in fluorescence-guided surgery. In breast cancer surgery, ICG is considered the most promising novel tracer for sentinel lymph node biopsy (SLNB).^1,2^ Recent breast cancer practice guidelines from Europe, Japan, and China have identified ICG as a safe alternative to radioactive colloids (^99m^Tc-sulfur colloid or ^99m^Tc-phytate) or blue dye (e.g., patent blue or methylene blue).^3–5^ However, US Food and Drug Administration (FDA) approval for its use in breast cancer sentinel lymph node biopsy, including both the medication and associated medical devices, remains lacking.

ICG has a high molecular weight and exhibits hydrophilic and lipophilic properties simultaneously.^6^ It has been known for years that, without dilution, ICG particles tend to form H-aggregates, a phenomenon also known as quenching, which affects optical fluorescence in terms of emission wavelength and intensity.^6–10^ Since ICG was originally developed for hepatic function measurement, most generic formulations use distilled water as the stock solvent, which is suboptimal for emissive fluorescence imaging. This complicates maintaining imaging consistency, impacts the development of imaging devices, and hinders the reproducibility of relevant research. Owing to ICG’s lipophilic properties and molecular polarity, colloids or organic solvents, such as albumin or ethanol, have been used as solvents to prevent ICG aggregation and enhance the fluorescence SBR.^6–11^

Currently, very few colloidal solutions are clinically available. The most common ones are human serum albumin (HSA) and 6% hydroxyethyl starch (Voluven®). It has been reported that ICG particles bind to serum proteins, such as albumin, stabilizing their molecular structure and sustaining enhanced fluorescence emission.^6,7^ Consequently, numerous studies have investigated the use of HSA solution as a solvent for ICG in lymphatic mapping procedures and have concluded that using albumin alone with ICG may provide additional benefits.^8,9^ However, two clinical trials have refuted this hypothesis, and a subsequent meta-analysis suggested that diluted ICG without conjugation of HSA could provide similar benefits with greater cost-efficiency.^1,9,10^ Following the publication of these landmark papers, further pursuit of adjuvant solutions for optimizing ICG fluorescence has ceased.

Our translational study, initiated in 2022, first investigated the properties of Voluven®-assisted ICG, in which ICG particles were effectively protected from forming H-aggregates by Voluven®. On the basis of this hypothesis, we proposed a new protocol using Voluven®-assisted ICG for sentinel lymph node biopsy (SLNB) in patients with breast cancer, offering improved fluorescence guidance and better mapping image quality in the first part of the clinical trial.^11^ This paper reports the second part of the clinical trial, which is a dose-optimization trial evaluating the optimal proportion of ICG:Voluven for breast cancer sentinel lymph node mapping.

## Patients and Methods

### Dose-Image Optimization Trial Design

The clinical trial was approved by the Institutional Review Board (IRB) of National Taiwan University Hospital Hsin-Chu Branch in accordance with the ethical standards of the 1975 Declaration of Helsinki, and was registered at clinicaltrials.gov (NCT05365204). The off-label use of ICG and Voluven® for breast cancer SLN mapping was evaluated and approved by the IRB (no. 111-009-F). The trial was designed as a prospective dose-adjustment trial with written inform consent.

**Fig. 1.**
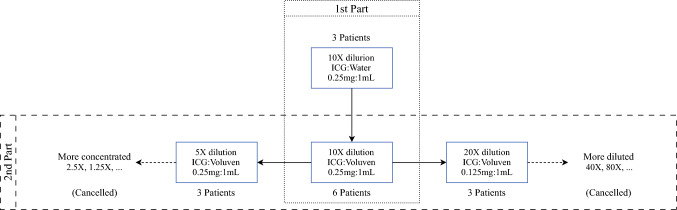
The diagram of study design and patient grouping; the first part of the study compared using water and Voluven® as solvent,^11^ while the second part of the study tested different dilution ratio for best fluorescence imaging; the “more diluted” groups and the “more concentrated” groups were cancelled due to inferior results of the 20× dilution group and 5× dilution group

The “default concentration” of ICG was set at 0.25 mg ICG in 1 mL Voluven® (0.25mg/1mL ICG:Voluven) on the basis of our previous basic research.^11^ The patients were invited in groups of three for each new concentration, and the new concentration group was discontinued if the outcome (see endpoints below) was obviously worse than the previous concentration. Figure 1 illustrates the patient grouping diagram. The first part of the study assessed the benefits of using Voluven® as a solvent instead of water, while the second part explored different concentrations in both diluted and concentrated forms. This paper presents the results of the second part of the clinical trial. The inclusion criteria were: (1) freshly diagnosed early breast cancer eligible for SLNB procedure, (2) no known allergy to ICG or blue dye, and (3) female participants older than 20 years.

### Preparation and Dilution Protocol of the ICG:Voluven Solution

The clinically used ICG solution was Diagnogreen® 25 mg/vial (Daiichi Sankyo Propharma Co. Ltd., Japan). Voluven® (1000 mL/bag) was obtained from Fresenius Kabi Deutschland GmbH, Germany. During the preparation of the ICG solution, the stock distilled water ampoule of Diagnogreen® was not used to avoid the formation of ICG H-aggregates. Instead, 10 mL of Voluven® was used to dissolve the powdered ICG, with manual shaking of the solution for at least 20 s. After dissolution and emulsification, the solution was 25 mg ICG in 10 mL of Voluven®. The dilution ratio was calculated on the basis of this concentration.

**Fig. 2:**
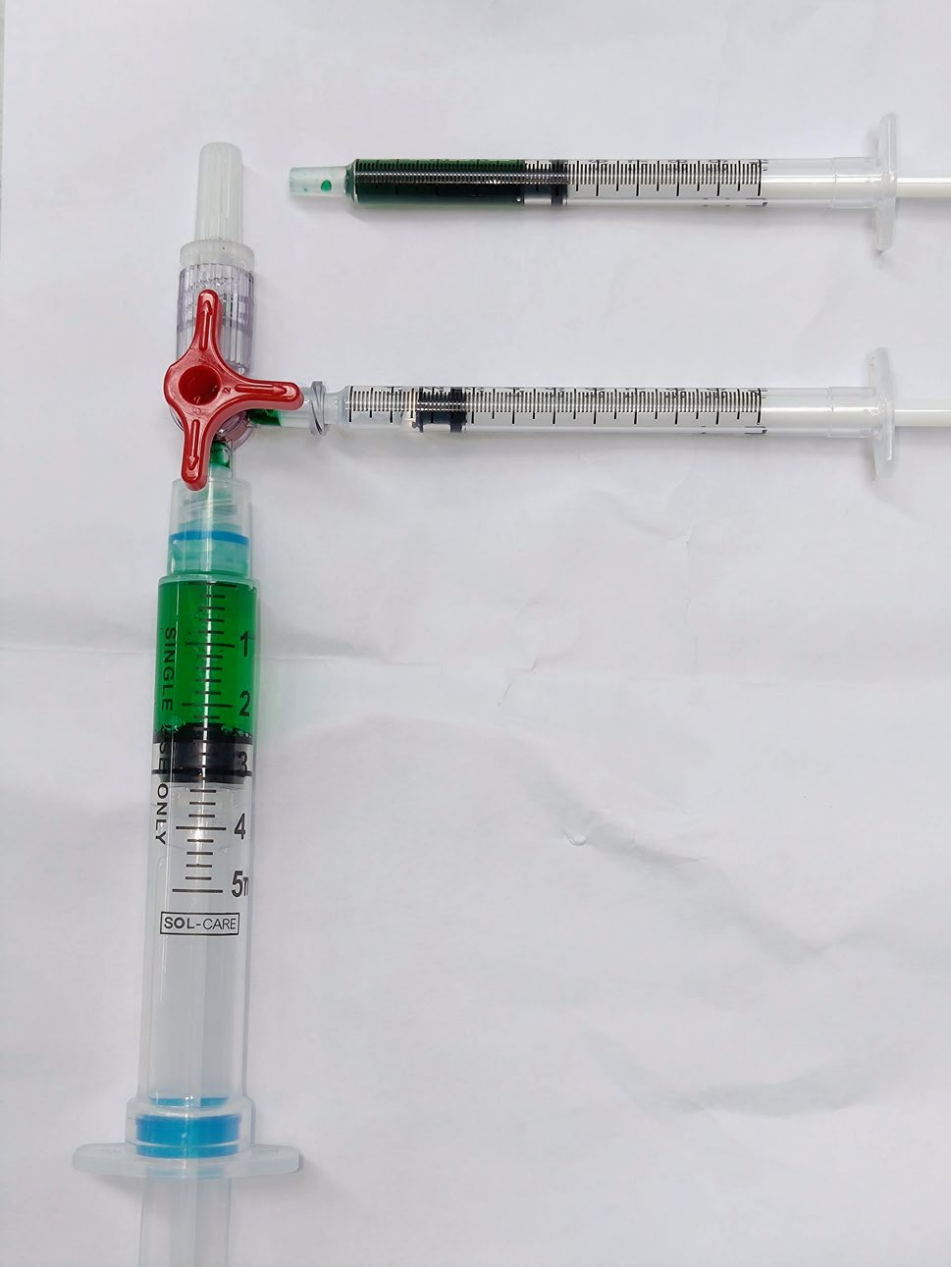
Three-way connector technique was used to prepare different concentrations of the ICG:Voluven solution; Voluven® (10 mL) was used to dissolve powdered ICG instead of stock distilled water; the reconstituted ICG:Voluven solution (2.5 mg/mL) was withdrawn from the vial and transferred into a 5 mL syringe via the connector; a calculated volume of diluent Voluven® solution was then added, for example, to achieve a 10× dilution, 0.5 mL of ICG:Voluven (2.5 mg/mL) was combined with 4.5 mL of Voluven®; this technique minimizes contamination by preventing spreading or direct manual contact with the contrast agent

Further dilution was performed on the operating table as soon as possible. The emulsified solution (25 mg ICG in 10 mL Voluven®) was drawn into a 1 mL syringe. A three-way connector was used to connect the 5 mL empty syringe for receiving the final mixture (see Fig. 2). For example, a 10× diluted solution is prepared by injecting 0.5 mL of the emulsified solution (25 mg ICG in 10 mL Voluven®) along with 4.5 mL of Voluven® into the receiving syringe, followed by manual shaking. This results in a 0.25 mg/1 mL ICG:Voluven solution. The other solution with different dilution ratios were prepared with the same protocol.

### Repetitive Injection-Observation Protocol

The injection protocol was adhered to achieve the best mapping imaging quality.^11^ It includes the following key points: (1) stable fixation of the injection needle tip subareolarly, (2) slow injection, administering 0.5 mL of the prepared solution each time, and (3) once subcutaneous lymphatic fluorescence has developed, administering a further 0.5 mL of the solution. These protocols aim to avoid repetitive skin punctures, minimize the subcutaneous water-dissection effect, allow the lymphatic system to uptake the medication gradually, and ensure enough fluorescent particles are drained to the axilla.

The injection procedure was performed with the operating room (OR) lights turned off and was observed using the Stryker SPY-PHI (Stryker Corporation, Kalamazoo, MI; portable handheld NIR imaging system). The timing of the first injection, each additional 0.5 mL injection, the visualization of fluorescent lymphatics, and the time when the fluorescence reached the axilla were all recorded intraoperatively. Light palpation could be performed if the drainage seemed to be stationary, but milking to the lymphatics was prohibited.

### Surgical Procedure and Measurement of Areola-to-Axillary Times and Signal-to-Background Ratios

The surgery was performed by a single surgeon who regularly performs ICG fluorescence-guided SLNB for breast cancer. After the injection of the ICG solution, peri-tumoral blue dye was injected under ultrasound guidance, forming a dual-tracer SLNB technique. The procedure usually starts 5–10 min after the injections. The SLNs were retrieved by tracing the blue duct or fluorescent duct and labeled with sequential numbers. If the sequence of the lymph nodes could not be determined by the lymphatic duct, it was determined by the distance from the breast.

Since it is a known fact that ICG-SLNB tends to retrieve more lymph nodes, the surgeon was allowed to excise a maximum of three sentinel lymph nodes to avoid over-dissection.^1,12^ If there were more sequential fluorescent lymph nodes and the retrieved SLNs were suspected to be metastatic, frozen sections of the SLNs were sent for analysis. Further dissection of the lymphatics would not be performed if the result was negative. It is worth mentioning that the sequence of SLNs was stratified by visual inspection and manual palpation, thus it is possible that more than one lymph node is contained in each labeled specimen, as shown in the pathology reports.

After retrieving the SLNs, the specimen was placed in a metal kidney dish to provide a standardized background. Fluorescent photographs were then captured using the Stryker SPY-PHI system in near-infrared (NIR) grayscale mode. The SBRs were measured using ImageJ software (bundled with Java 1.8.0_172). Data visualization and plotting were performed using R version 4.2.2 (R Foundation, Vienna, Austria).

### Endpoints

The primary endpoint was the number and the SBR of retrieved lymph nodes. Other endpoints included the areola-to-axilla traveling time (AAT), defined as the time from the development of visible subcutaneous lymphatic fluorescence to the fluorescence signal reaching the axillary fascia, the presence of blue dye in the SLNs, any inadvertent events during the procedure, and any adverse effects reported.

## Results

### Patient Characteristics

From April 2022 to June 2023, 12 consecutive patients were enrolled in the second part of the study. Table 1 presents the characteristics and oncological information of the recruited participants. All participants were female, diagnosed with breast cancer, and clinically node negative. Postoperative staging ranged from pT1 to pT2, pN0 to pN1. Ages ranged from 36 to 83 years, and body mass index (BMI) ranged from 15.1 to 32.0 kg/m^2^.

**Table 1:** Patient characteristics and oncological stagings

Case	Age	BMI	Type of invasive carcinoma	Grade	ER	PR	HER-2 score	Ki-67 index	Tumor size (mm)	Pathological staging
10 × 01	64	19.6	NST	I	> 95%	> 95%	0/3+	10–15%	10	pT1b pN0
10 × 02	36	20.9	Mixed lobular and NST	II	> 95%	> 95%	0/3+	5–30%	20	pT1c pN1a
10 × 03	80	22.2	Solid papillary (multifoci)	II	> 95%	> 95%	0/3+	15–20%	9	mpT1b pN0
10 × 04	43	23.8	Tubular	I	> 95%	> 95%	1+/3+	10–20%	8	pT1b pN0
10 × 05	65	15.1	NST	III	> 95%	30%	1+/3+	40–70%	22	pT2 pN0
10 × 06	50	34.9	NST	III	3%	0%	1+/3+	60–80%	28	pT1 pN0
20 × 01	43	22.2	Mucinous	I	> 95%	> 95%	0/3+	10–30%	23	pT2 pN0
20 × 02	83	21.8	NST	I	> 95%	60%	0/3+	5–20%	20	pT1c pN0
20 × 03	74	32.0	Papillary	II	> 95%	30%	0/3+	15%	6	pT1b pN0
05 × 01	73	27.6	NST (multifoci)	II	> 95%	> 95%	0/3+	40–70%	15	mpT1c pN0
05 × 02	59	24.3	NST	II	> 95%	> 95%	0/3+	3–20%	18	pT1c pN0
05x03	64	23.6	NST	III	>95%	1%	0/3+	30–70%	13	pT1c pN1mi

*BMI* body mass index, *ER* estrogen receptor, *PR* progesterone receptor, *NST* no special type, *HER-2* human epidermal growth factor receptor 2

### Stepwise Tests of Different Concentrations

The injection data and fluorescence properties were presented in Table 2; six patients were assigned to “10× group” using the default concentration (0.25 mg/mL ICG:Voluven) for testing the stability of default concentration. We further evaluated different concentrations in a stepwise manner.

**Table 2:** Fluorescence mapping and status of sentinel lymph nodes information

								SLN1^b^			SLN2			SLN3		
Case	Dilution rate (×)^a^	Volume (mL)	Total ICG amount (μg)	Observe time (s)	Infusion rate (μl/s)	Drain initiation time(s)	AAT (s)	SBR	Blue	Status (pos/total)	SBR	Blue	Status (pos/total)	SBR	Blue	Status (pos/total)
10 × 01	10	2	500	47	42.6	16	31	57.6	Yes	0/1	97.2	No	0/1	75.7	No	0/1
10 × 02	10	1.5	375	33	45.5	12	21	90.9	Yes	1/1	27.9	No	0/1	(N/A)		
10 × 03	10	2	500	70	28.6	14	56	> 255	Yes	0/1	> 255	Yes	0/1	50.7	No	0/1
10 × 04	10	2	500	156	12.8	59	97	159.2	Yes	0/1	70.1	Yes	0/1	94.3	No	0/1
10 × 05	10	2	500	354	5.6	221	133	141.2	Yes	0/1	80.8	No	0/2	57.6	No	0/1
10 × 06	10	2.5	625	265	9.4	169	96	113.6	Yes	0/1	109.8	Yes	0/1	70.9	No	0/1
20 × 01	20	3.5	438	159	22.0	100	59	39.1	Yes	0/1	28.8	No	0/1	(N/A)		
20 × 02	20	4.5	562	274	16.4	242	32	5.3	No	0/1	1.2	Yes	0/1	(N/A)		
20 × 03	20	2.5	312	107	23.4	65	42	73.9	No	0/1	98.4	No	0/1	60.4	No	0/4
05 × 01	5	1.5	750	300	5.0	99	201	26.3	Yes	0/1	19.8	No	0/1	(N/A)		
05 × 02	5	2.0	1000	404	2.0	325	79	2.7	Yes	0/1	(N/A)			(N/A)		
05 × 03	5	1.5	750	234	1.5	81	153	74.0	No	0/2	133.2	Yes	1/1	26.0	Yes	0/2

^a^Dilution rate 5× = 0.5 mg ICG in 1 mL Voluven®; 10× = 0.25 mg ICG in 1 mL Voluven®; 20× = 0.125 mg ICG in 1 mL Voluven®.

^b^The sequence of SLNs was stratified by visual inspection and manual palpation, so it is possible that more than one lymph node is contained in each labeled specimen.

*AAT* areolar-to-axilla traveling time, *SLN* sentinel lymph node, *BMI* body mass index, *OE* overexposure, *N/A* not applicable

First, we tested the “diluted arm,” involving three patients in the “20× group,” which used 0.125 mg/mL ICG:Voluven. The fluorescence SBR of the brightest node in this group was lower than in the default 10× group (median, range = 39.1, 5.3–98.4 in the 20× group versus 127.4, 90.9–256.0 in the 10× group). However, the total amount of ICG was similar (median, range = 437.5 µg, 312.5–562.5 µg versus 500 µg, 375–625 µg in the 10× group). The number of sequentially retrieved sentinel nodes was also fewer in this group compared with the default group, with two out of three patients having only two sentinel lymph nodes (SLNs) retrieved. Due to the inferior outcomes, this arm was discontinued.

Next, we tested the “concentrated arm,” with three patients in the “5× group,” using 0.5 mg/mL ICG:Voluven. The fluorescence SBR of the brightest node in this group was also lower than in the default 10× group (median, range = 26.3, 2.7–133.2 in the 5× group versus 127.4, 90.9–256.0 in the 10× group). The total injected amount of ICG was higher (median, range = 750.0 µg, 750.0–1000 µg versus 500 µg, 375–625 µg in the 10× group). Similarly, the number of sequentially retrieved sentinel nodes was fewer, with one patient having only one SLN retrieved, and another patient having only two SLNs retrieved. Consequently, the “concentrated arm” was also discontinued.

### Fluorescence Properties of Injection Process

**Fig. 3:**
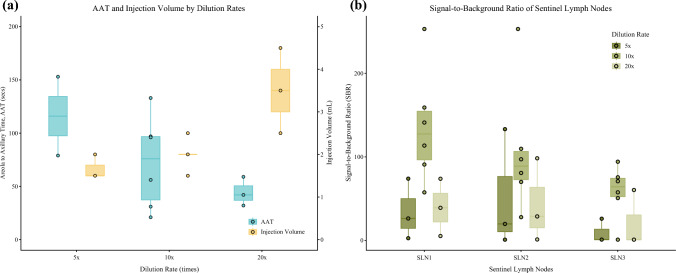
**A** AATs and injection volumes under different concentrations of ICG:Voluven; **B** signal-to-background ratios of the sequential SLNs; SBRs of the overexposed LNs were plotted as 256:1, while that of the undetected LNs were plotted as 1:1 (no contrast) for better visualization

The areola-to-axillary traveling times (AATs) and injection volumes are plotted in Fig. 3A. We observed that as the solution is more diluted with Voluven®, the AAT is shorter. The mean total injection volumes for the 5×, 10×, and 20× groups were 1.67 mL, 2.0 mL, and 3.5 mL, respectively, while the mean AATs were 144.3 s, 72.3 s, and 44.3 s, respectively.

The SBRs of the retrieved sequential SLNs are plotted in Fig. 3B. The overall trend shows lower SBRs of the SLNs for the 5× group and the 20× group compared with the default 10× group. Only patients from the 10× group exhibit overexposure lymph node pictures taken with Stryker SPY-PHI. Two patients from the 5× group and two patients from the 20× group had no fluorescent SLN3 and therefore the SBR was coded as 1:1 for visualization.

### Intraoperative Events, Postoperative Events, and Follow-Up Outcomes

There were no complications observed intraoperatively, and no relevant complaints were reported postoperatively. The first patient (of the default concentration group) experienced inadvertent leakage of the ICG:Voluven solution from the nipple during the injection process. We immediately adjusted the needle tip, and the procedure was not affected. No more nipple leakage happened to the rest of the participants.

Follow-up data were recorded until June 2024, and no axillary recurrence or lymphedema was observed. Additionally, there was no report of abnormal scarring or tattooing of the nipple–areola complex during the follow-up period.

## Discussion

This trial reveals insights regarding the use of ICG in Voluven® as a fluorescent contrast agent to visualize lymphatic ducts and lymph nodes. First, using Voluven® as a solvent for ICG in lymphatic fluorescence imaging is a safe and effective method. Second, even though the total amount of ICG used is similar, it does not result in consistent SBR for fluorescence imaging across diverse dilution ratios. Third, the proportion between ICG and the solvent, Voluven®, in this trial affects not only the SBR, but also the areola-to-axilla traveling time (AAT). Fourth, the default 10× group (0.25 mg/mL ICG:Voluven) shows the best SBR, resulting in the retrieval of more SLNs. It is worth mentioning that when Voluven® is used as a solvent, it can highly enhance the SBRs; however, this advantage would significantly diminish if the concentration is not precisely controlled.

There are already many proposed injection protocols for breast cancer SLNB.^8,13,14^ Most injection protocols only emphasize the amount of ICG. Some research tried to investigate using albumin as a solvent to enhance ICG fluorescence.^8,9^ However, the injection details, such as injection point, infusion speed, and different ICG:solvent proportion were not evaluated delicately. Most of the research uses regular tracers simultaneously, which is probably due to the lack of FDA approval of ICG fluorescence SLNB, making it difficult to evaluate the actual usability of ICG as a single primary tracer. In addition, most clinical research does not define the SBR of the targets, which is crucial in fluorescence imaging.

As aforementioned, ICG’s photophysical properties vary between monomer form and H-aggregates, while ICG tends to form H-aggregates using water as solvent without appropriate dilution. The monomer form of ICG is the major emissive form with high fluorescence brightness when used as NIR contrast as compared with the aggregation state. However, the monomer form dominates only in low concentrations.^6^ In our last study, both the absorption spectrum and emission intensity indicated that using Voluven® as a solvent can effectively prevent ICG from aggregation-caused fluorescence quenching, and thereby maintains ICG in monomer emissive form even in elevated concentrations.^11^

**Fig. 4:**
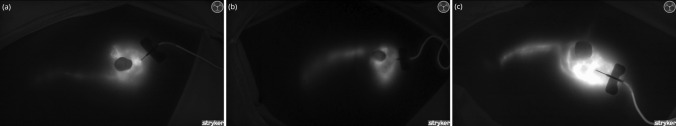
Example fluorescence images of the subcutaneous lymphatics of the breasts from different concentration groups; **A** a 59-year-old woman in the 5× concentration group showed uneven distribution of the fluorescence, which indicates incomplete emulsification and quenching of the ICG particles; **B** a 51-year-old woman in the 10× concentration group showed even subareolar fluorescence with thicker subcutaneous lymphatics, which could be a volume expansion effect of Voluven®; **C** a 43-year-old woman in the 20× concentration group showed great volume expansion effect of the lymphatics, but subcutaneous dissection effect around the injection site

 To improve the bioavailability of subcutaneous drugs, we must revisit how the lymphatic system governs their absorption and transport. It has been reported that some factors affect the pharmacokinetics and pharmacodynamics of subcutaneous drug administration, such as anatomical architecture of the injection site, injection depth, and formulation of the solution.^15,16^ Current theories in subcutaneous biomolecule transportation suggest that molecules with low molecular weights (< 16 kDa) can be absorbed through capillaries, while high molecular weight particles travel through the lymphatic system.^15^ ICG’s molecular weight, in its monomer form, is approximately 0.775 kDa, which is too low to stimulate the opening of lymphatic endothelial cells. When conjugated with albumin (66–67 kDa), or in this study, Voluven® (average molecular weight of 6-HES is around 130 kDa), the higher molecular weight stimulates the opening of endothelial cells and facilitates the entry of substances into the lymphatic system.^17–21^ This could also explain why, in this trial, the 20× diluted group showed a slower initiation of lymphatic drainage but excelled in drainage speed (AATs). It takes time for endothelial cells to open, while the uptake of the colloidal solution is rapid once the effect takes place. However, a more diluted ICG:Voluven solution requires a higher total volume to be injected, which could cause subcutaneous water dissection and spread the fluorescent solution toward undesired directions (Fig. 4).

Wrapping the above points, the theoretically optimal adjuvant solvent for ICG lymphatic mapping should (1) be able to maintain ICG monomer form at high concentration during preparation, (2) have high molecular weight to stimulate lymphatic uptake, and (3) have the ability to deliver ICG monomer particles to the desired position. This dose-optimization trial using ICG:Voluven provides a solution to the former two unmet needs. Our previous translational study also reported promising results for the third point using 4T1 mouse model, which could be explained with better EPR effect when ICG conjugated with 6-HES.^11,22,23^ Further clinical translation will be investigated.

On the basis of our previous translational study and this dose-optimization trial, we propose the following optimal injection protocol for breast cancer sentinel lymph node mapping using ICG:Voluven as follows: 0.25 mg:1 mL ICG:Voluven, administered via a single-point subcutaneous injection at the areola, with a slow infusion rate and a total volume 1.5–2.5 mL. This novel injection protocol reduces the dosage of ICG (maximum 0.625 mg in this study) while providing better contrast on the fluorescence images. Because of the dilution and low cost of Voluven®, the cost of ICG:Voluven (0.25 mg:1 mL) was low, estimated at approximately 1.5 US dollars per patient when excluding the cost of the imaging device.

This study has several limitations, including a small sample size due to its phase I trial design and potential selection bias arising from the serial participant invitation process. These limitations may be addressed in future studies, such as phase II–III trials. In future clinical trials of a similar nature, it will be essential to integrate drug improvements, optimized injection techniques, and calibrated adjustments to medical imaging devices to achieve reliable and reproducible results.
